# Migration of a Retained Epicardial Pacing Wire Into the External Carotid Artery

**DOI:** 10.1016/j.jaccas.2025.103616

**Published:** 2025-06-11

**Authors:** Oluwakorede Akele, Frank Rosell, Nathan Cornish, Jonathan Scheiner, Mark Raden, Rohit Shahani

**Affiliations:** a Northwell Health, New Hyde Park, New York, USA; b Department of Cardiothoracic Surgery, Staten Island University Hospital Heart Institute, Staten Island, New York, USA; c Department of Radiology, Staten Island University Hospital, Staten Island, New York USA

**Keywords:** endovascular intervention, perioperative cardiac arrhythmias management, temporary epicardial pacing wires, wire migration

## Abstract

**Background:**

Temporary epicardial pacing wires (TEPWs) are commonly used for postoperative arrhythmia management in cardiac surgery. Although generally safe, they can cause rare complications, including migration years after placement.

**Case Summary:**

A 59-year-old man with a history of cardiac arrest and acute myocardial infarction requiring urgent coronary artery bypass grafting 2 years prior presented with new-onset dysphagia and odynophagia. Full workup and imaging revealed a migrated TEPW embedded in the right external carotid artery. The wire was successfully removed using advanced endovascular techniques, providing immediate symptom relief.

**Discussion:**

This rare case of a retained TEPW highlights the importance of “thinking outside the box.” Combinatorial analysis, which combines medical knowledge with comprehensive data from numerous simple observations, tests, and procedures, guided us to the optimal treatment for our patient.

**Take-Home Message:**

Clinicians should maintain vigilance for delayed TEPW complications and consider alternatives that reduce long-term risks associated with retained wires.

## Visual Summary

**Figure:**
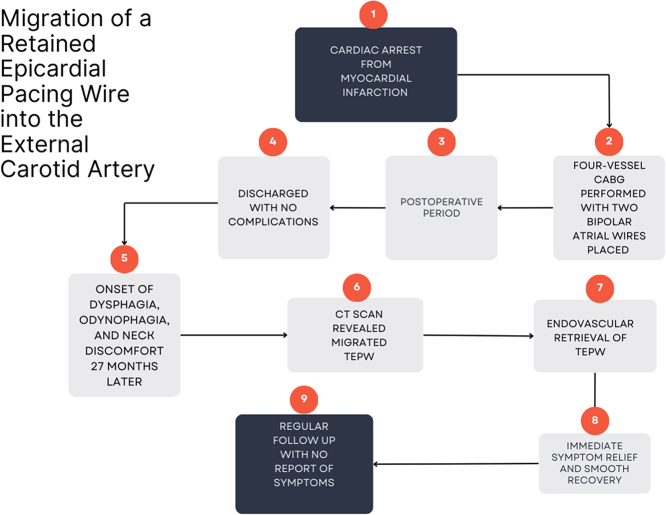

## History of Presentation

**Figure 1:**
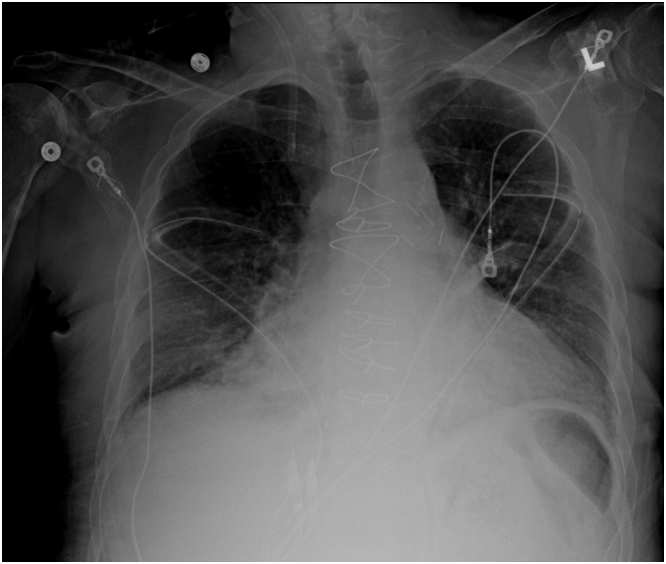
Postoperative CXR With the TEPW In Situ Postoperative chest x-ray of the patient showing no evidence of migrated temporary epicardial pacing wires.

A 59-year-old man with a 4-vessel coronary artery bypass grafting presented with a 3-week history of sharp right neck pain, odynophagia, and mild dysphagia. Twenty-seven months before this presentation, he presented with cardiac arrest from an acute myocardial infarction, was resuscitated, and was treated in the intensive care unit for septic shock and atrial fibrillation. Echocardiography showed an ejection fraction of 40% to 45% with wall motion abnormalities. After partial recovery, he underwent a 4-vessel coronary artery bypass grafting for severe triple-vessel disease, with 2 bipolar atrial wires placed during surgery. Because he was anticoagulated early postoperatively, his pacing wires were cut and retracted after gentle traction failed. He gradually recovered, and the patient's chest x-ray showed no migrated wires (Figure 1). He was discharged on postoperative day 11. At follow-up, he reported a complete resolution of cardiac symptoms. On the recent physical examination, there was new mild tenderness on palpation of the right side of the neck, and no masses were palpated.

**Take-Home Messages**

• This case highlights the long-term risks of retained TEPWs, including rare but severe complications such as wire migration into critical vascular structures, which can manifest unexpectedly years after surgery.
• Adopting strategic follow-up measures alongside emerging technologies like bioresorbable wires has the potential to enhance outcomes. Additionally, this case highlights the need for a transition toward more innovative and conservative management approaches, fostering safer practices that could profoundly improve patient outcomes in cardiology.

## Medical History

The patient had a medical history of severe peripheral arterial disease status after bilateral transmetatarsal amputations, end-stage renal disease on hemodialysis through a left forearm arteriovenous fistula, uncontrolled type 2 diabetes mellitus (hemoglobin A_1c_: 8.7%), coronary artery disease, 4-vessel coronary artery bypass graft, and hypertension.

## Differential Diagnosis

Possible etiologies to explain his symptomatology included neuralgia, muscle pain, or arthritic changes of the larynx.

## Investigations

**Figure 2:**
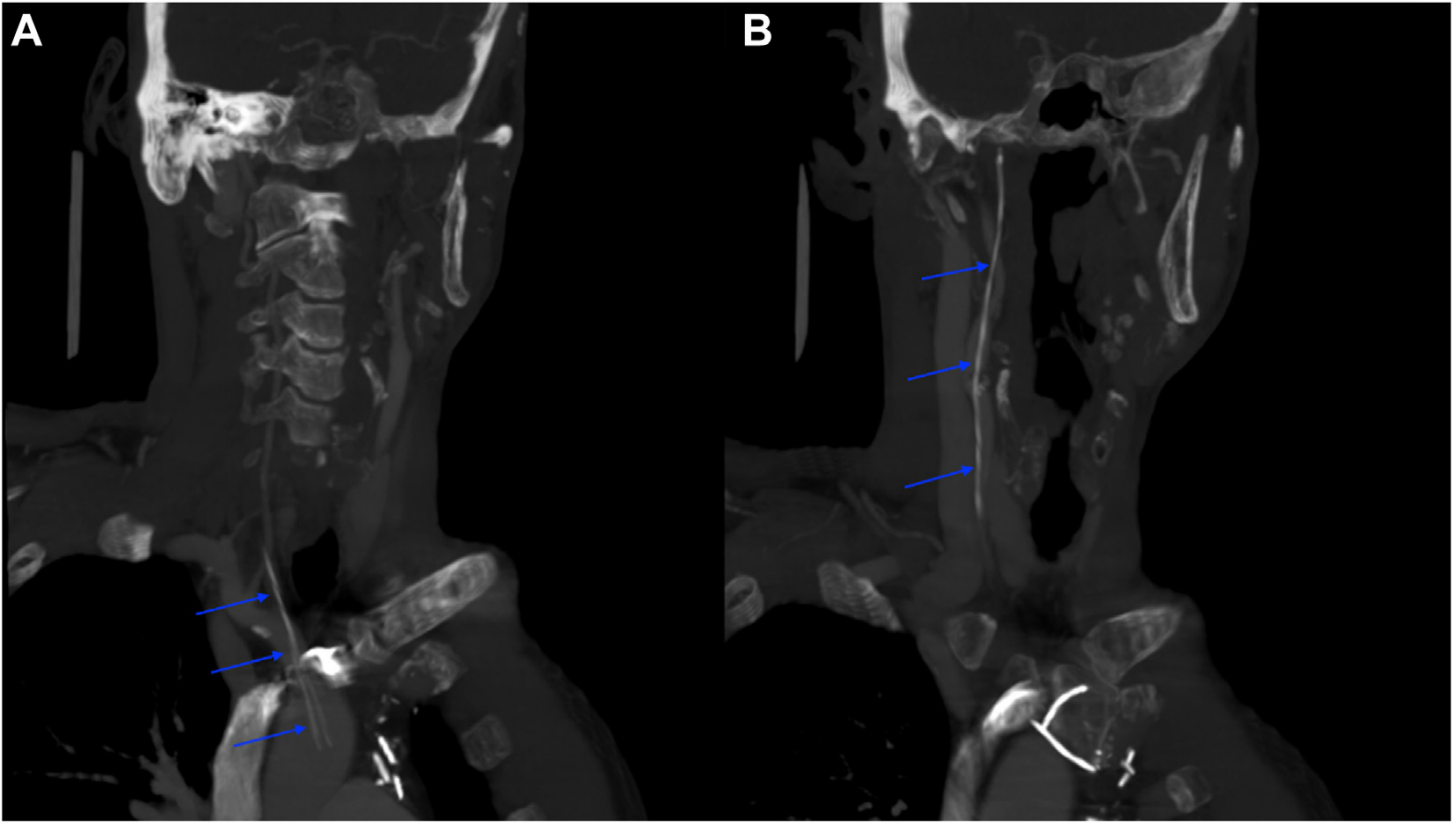
CT Neck Demonstrating the TEPW Migration Into Carotid Artery (A) Computed tomography of the neck with intravenous contrast showing the retained temporary epicardial pacing wire (TEPW) coursing through the brachiocephalic artery into the right common carotid artery, indicated by the blue arrows. (B) The same scan shows the retained TEPW extending from the right common artery, as indicated by the blue arrows.

**Figure 3:**
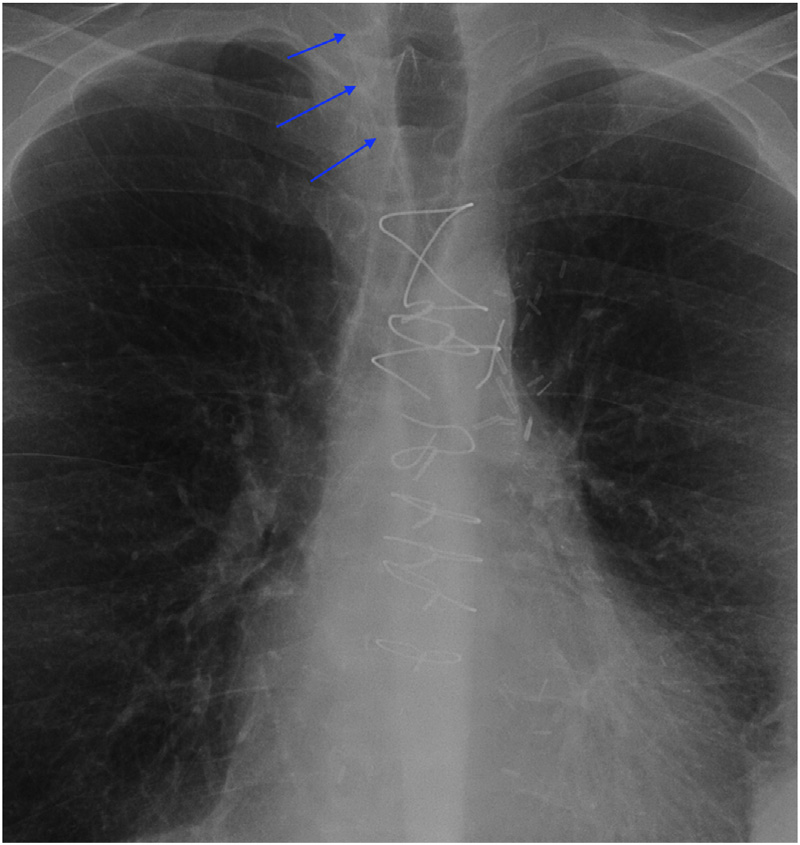
Chest X-Ray Demonstrating the TEPW Migration Into Ascending Aorta and Carotid Artery Chest x-ray 5 months after the operation showing retained temporary epicardial pacing wire coursing along the ascending aorta into the right common carotid artery, indicated by the blue arrows.

As part of his ongoing workup, he underwent a flexible laryngoscopy, which was unrevealing. The patient had a computed tomography of the neck with intravenous contrast, which revealed a metal catheter wire extending into the right external carotid artery at the level of the pterygoid musculature (Figures 2A and 2B). These findings were different from prior imaging. On discussion with the patient and cardiothoracic surgeon, it was determined that this was most likely the retained epicardial wire, based on a retrospective review of previous serial chest x-rays the patient had after discharge (Figure 3).

## Management

**Figure 4:**
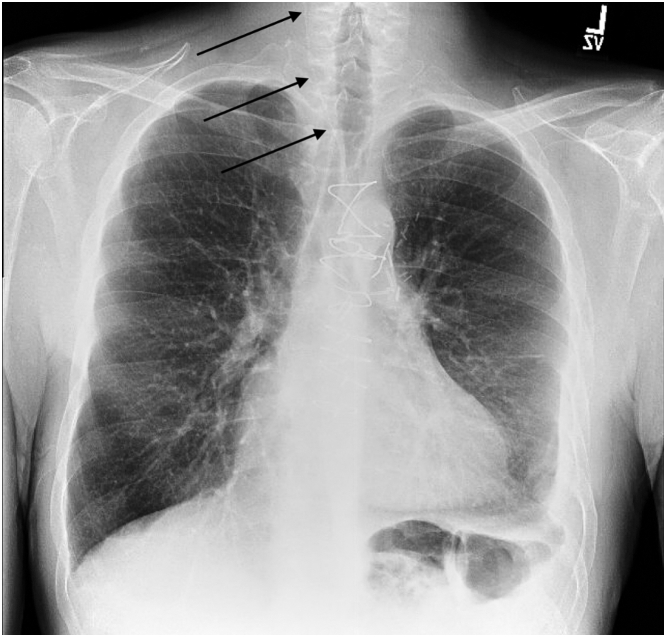
Preoperative Chest X-Ray Showing Migrated the TEPW in the Aorta and Carotid Artery Chest x-ray showing the retained temporary epicardial pacing wire tracing from the aorta, coursing along the aorta into the right common carotid artery indicated by the black arrows, taken during preoperative workup for wire retrieval.

**Figure 5:**
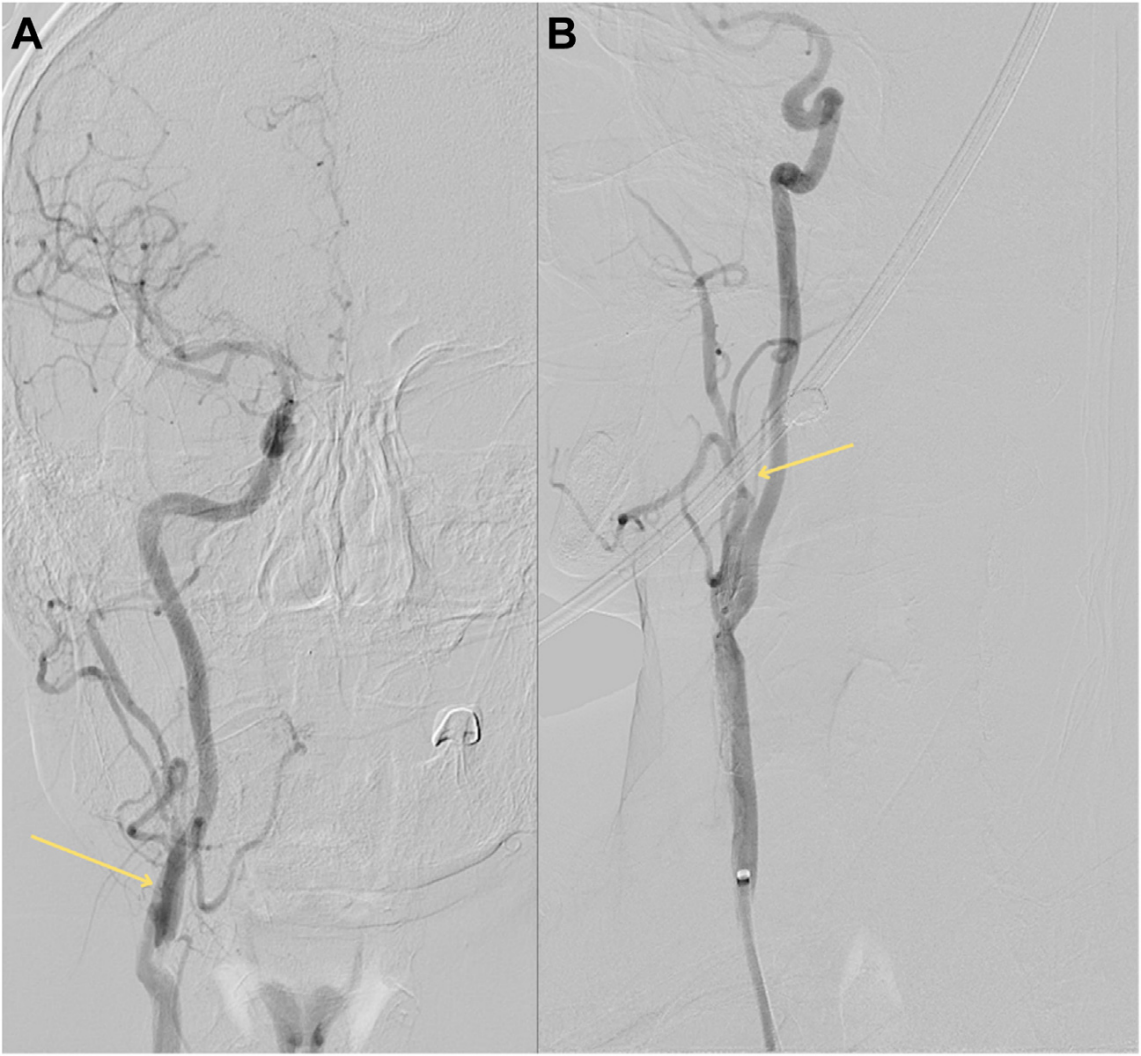
Cerebral Angiogram Demonstrating the TEPW and Carotid Artery Spasm Post-Removal (A) Cerebral angiogram of the right common carotid artery that shows the retained epicardial wire coiled up, creating a bulge, indicated by the yellow arrow. (B) The same scan shows a spasm of the external carotid artery after removing the wires, indicated by the yellow arrow.

The case was discussed with our interventional radiology colleagues, and the decision was made to remove the wires. After perioperative imaging (Figure 4), the patient underwent endovascular wire removal under conscious sedation. The left common femoral artery was accessed using the Seldinger technique, and a 6-F-long sheath was advanced into the aortic arch, where the wires were identified. A 30-mm gooseneck snare was used, but only 1 wire was initially retrieved because of significant resistance. Fluoroscopy showed the remaining wire balled up in the right external carotid artery and extending into the right common carotid artery (Figure 5A). A 10-mm cloverleaf snare successfully removed the remaining wire.

## Outcome and Follow-Up

**Figure 6:**
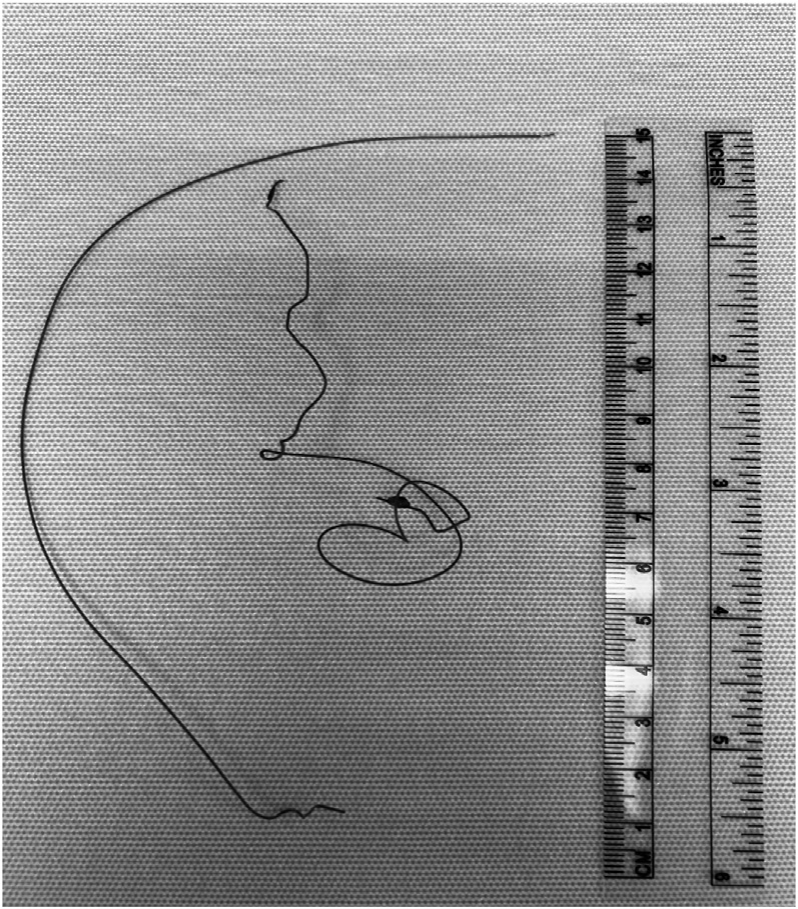
Retrieved Bipolar Atrial TEPWs Post-Extraction Image showing the 2 bipolar atrial wires retrieved from the patient.

After the procedure, the patient reported immediate alleviation of pain. Subsequent angiography confirmed the presence of spasms in the proximal external carotid artery; however, it revealed no abnormalities in the internal carotid artery or intracranial branches, suggesting that the intervention did not adversely affect these areas (Figure 5B). In the postoperative period, the patient's recovery was smooth and uneventful. Follow-up assessments showed that he maintained good health, with no recurrence of the initial symptoms or new complications related to the procedure (Figure 6).

## Discussion

Temporary epicardial pacing wires (TEPWs) have been used since the 1960s and are commonly used for managing dysrhythmias and identifying and diagnosing perioperative electrical alterations.^1^ The use of TEPWs has led to lower morbidity and mortality, and <0.04% of patients suffer a significant complication after TEPW removal. The complication risk increases if patients undergo redo cardiac surgery or take anticoagulants, because this can cause cardiac tamponade.^2^ After surgery, these wires are usually left in place and removed before discharge. When patients no longer need temporary pacing, the wires are removed. They should generally be removed before starting oral anticoagulation.^3^ TEPWs can be removed by gentle but purposeful traction^3^ or by cutting them flush with the skin so that the residual wire retracts into the tissue.^4^ However, the cutting option has its own unique set of complications.^4^

Although cardiothoracic surgeons perform the initial surgery, long-term follow-up often transitions to general cardiologists and relevant subspecialists. These clinicians are essential in recognizing and managing device-related complications, which may manifest months or even years postsurgery, as demonstrated in our case. This case exemplifies the complexities of such consequences and illustrates why awareness and education about these potential issues are vital for the medical community in general. Additionally, it demonstrates the critical value of interdisciplinary collaboration among cardiologists, cardiothoracic surgeons, and interventional radiologists to manage and, ideally, prevent these potentially serious complications.

Although prior cases have documented TEPW migrations involving the ascending aorta,^5^ our case is particularly notable because of the involvement of the carotid artery, which is extremely rare. Although it was spotted that the TEPW migrated, it was remarkable that the patient did not have symptoms until 2 years later. The presence of this migrated wire was not noted on previous chest x-rays. The involvement of the carotid artery can increase the risk of life-threatening complications such as stroke or arterial injury during retrieval. The prior case of TEPW migration to the carotid artery did not mention neck pain as a symptom.

The mechanism behind TEPW migration is not well understood. One hypothesis suggests that movement of the chest wall and diaphragm, along with changes in intrathoracic and intra-abdominal pressures, could cause direct perforation of the myocardium, leading to wire migration.^6^ There has been intense speculation regarding the mechanism of wire migration, especially when found in unexpected organs in the body. We postulated that in our patient, the migration pathway most likely involved direct erosion across the right atrium and then through the interatrial septum to the left side of the heart, which was then ejected from the left ventricle into the systemic arterial tree. Conversely, the wire could have gained access to the left heart through the right superior pulmonary vein or the roof of the right atrium, as we often place the wire close to that area during surgery.^7^

The routine placement of TEPWs remains a topic of debate, with no clear consensus among cardiac surgeons on whether they should be used universally or selectively. Recent studies have shown that routine placement of TEPWs plays a negligible role and ultimately leads to more complications.^8^ No preventive measures or guidelines delineate the monitoring of TEPWs. We propose periodic chest x-rays that would reveal the location of the TEPW and a computed tomography with contrast if the wires are not easily visualized on chest x-rays. Alternatives to TEPWs include temporary transvenous wire, an electrode attached to an esophageal probe, and transcutaneous electrodes.^9^ Patients should be stratified for the need for TEPWs using a comprehensive evaluation based on the predictors mentioned earlier. This approach would lower the risk of complications. The use of molybdenum TEPWs appears promising, because they are bioresorbable and dissolve over time, potentially decreasing complications.^10^ However, temporary transvenous wires are more prone to dislodgement and infection. Transcutaneous pacing and an esophageal probe require sedation, and TEPWs do not.

## Conclusions

This case represents a rare and not insignificant complication of TEPWs, highlighting the potential for adverse outcomes of retained TEPWs even years after cardiac surgery. The patient’s initial presentation of dysphagia and odynophagia, which led to the discovery of a migrated TEPW into the right external carotid artery, displays the unpredictable nature of TEPW complications. Although retrieval was successful, the delayed presentation of symptoms suggests that an earlier intervention might have prevented symptoms.

Clinicians must maintain heightened awareness regarding the long-term risk of retained TEPWs, especially in patients where wires cannot be easily removed. Regular follow-up and imaging in high-risk patients could identify complications like wire migration before becoming symptomatic. Additionally, the need for the routine placement of TEPWs should be carefully evaluated, with patient stratification based on specific risk factors to minimize unnecessary complications. For high-risk patients, alternative pacing methods mentioned should be considered.

This case encourages a shift toward more innovative management strategies, promoting safer practices that could significantly impact patient outcomes in the general cardiology setting. Emerging innovations such as bioresorbable materials like molybdenum offer promising alternatives that could significantly reduce long-term risk by eliminating the need for wire removal. These advances, alongside ongoing research into alternative pacing methods, may provide safe and more effective options for managing perioperative dysrhythmias in cardiac surgery patients. Future studies and consensus on the routine use of TEPWs are necessary to optimize patient outcomes and minimize the associated risks.

**Visual Summary:**
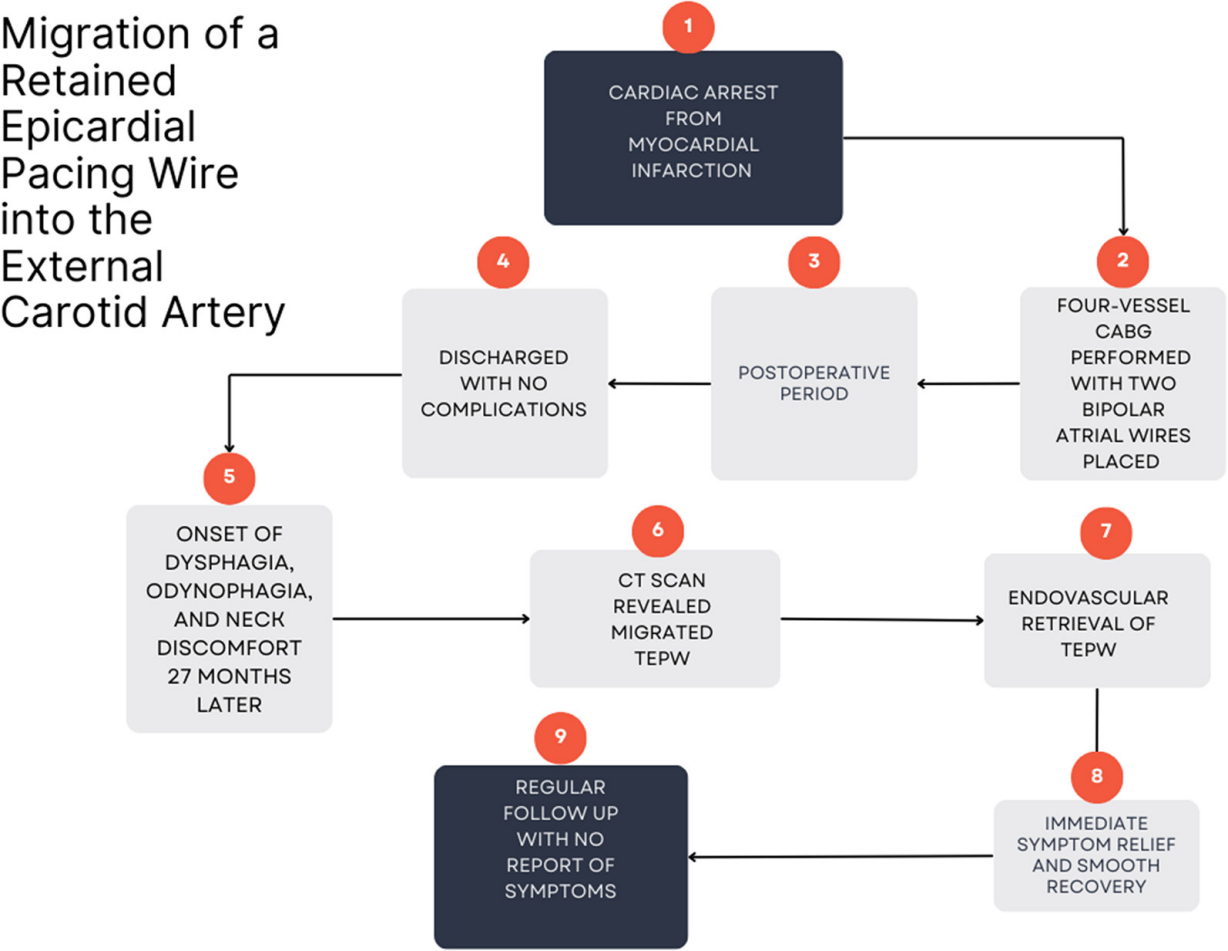
Timeline of the Case Study

## Funding Support and Author Disclosures

The authors have reported that they have no relationships relevant to the contents of this paper to disclose.
